# Experimental evidence that network topology can accelerate the spread of beneficial mutations

**DOI:** 10.1093/evlett/qrad047

**Published:** 2023-10-11

**Authors:** Partha Pratim Chakraborty, Louis R Nemzer, Rees Kassen

**Affiliations:** Department of Biology, University of Ottawa, Ottawa, ON, Canada; Department of Chemistry and Physics, Halmos College of Arts and Sciences, Nova Southeastern University, Ft. Lauderdale, FL, United States; Department of Biology, University of Ottawa, Ottawa, ON, Canada

**Keywords:** evolutionary graph theory, metapopulation topology, fixation dynamics, experimental evolution

## Abstract

Whether and how the spatial arrangement of a population influences adaptive evolution has puzzled evolutionary biologists. Theoretical models make conflicting predictions about the probability that a beneficial mutation will become fixed in a population for certain topologies like stars, in which “leaf” populations are connected through a central “hub.” To date, these predictions have not been evaluated under realistic experimental conditions. Here, we test the prediction that topology can change the dynamics of fixation both in vitro and in silico by tracking the frequency of a beneficial mutant under positive selection as it spreads through networks of different topologies. Our results provide empirical support that meta-population topology can increase the likelihood that a beneficial mutation spreads, broaden the conditions under which this phenomenon is thought to occur, and points the way toward using network topology to amplify the effects of weakly favored mutations under directed evolution in industrial applications.

The last two decades have seen the development of an empirically grounded theory of mutation-driven adaptation built on the assumption of a large, well-mixed population adapting through mutation to a uniform environment that remains constant in time (Bailey & Bataillon, 2016; Kassen, 2014; Lang et al., 2013; Lenski, 2017; Martin & Lenormand, 2008; Orr, 2005; Schoustra et al., 2009; Tenaillon et al., 2016). Yet most natural populations occupy environments that are far more ecologically complex than this theory assumes. One common form of ecological complexity is spatial structure, where a population is composed of a series of subpopulations connected by dispersal (also known as a metapopulation). How spatial structure impacts adaptive evolution, and in particular, the dynamics of substitution, in an otherwise uniform environment is not well understood.

By contrast, a rich theoretical literature exists on the effects of different forms of spatial structure on the population genetics of neutral variation (Hanski & Gilpin, 1997; Harrison & Hastings, 1996; Pannell & Charlesworth, 2000; Slatkin, 1985) and the interplay between migration and selection in adaptation (Blanquart et al., 2012; Guillaume, 2011; Yeaman & Otto, 2011). Empirical work has considered the impact of spatial structure through habitat fragmentation on trait evolution (Cheptou et al., 2017; Urban et al., 2008), how population subdivision modulates the extent of adaptive change (Bailey et al., 2021; Baym et al., 2016; Chao & Levin, 1981; Habets et al., 2006, 2007; Korona et al., 1994; Kryazhimskiy et al., 2012; Miralles et al., 1999; Nahum et al., 2015; Perfeito et al., 2007; Zhang et al., 2011) and community resilience (Limdi et al., 2018). Other work in microbiology has examined the emergence and fate of diversity in spatially structured environments associated with colony growth or biofilms (Borer et al., 2020; Celik Ozgen et al., 2018; France et al., 2018, 2019; Kerr et al., 2002; Nadell et al., 2010, 2016; Steenackers et al., 2016; Trubenová et al., 2022) but lacks explicit descriptions of spatial structure or conflates it with variation in conditions of growth that generate divergent selection (Chen & Kassen, 2020; Leale & Kassen, 2018). Missingare explicit tests of theory on how the spatial arrangement of populations in space impacts the dynamics of adaptive evolution, including the rate of spread of a beneficial mutation and the probability of fixation.

Here we explore the impact of network topology—the pattern of connectivity among subpopulations—on adaptive evolution by testing key predictions from two theoretical frameworks: one rooted in classic population genetics and the other in evolutionary graph theory (EGT). Population genetic models track the effects of migration and selection on gene frequencies, often under the simplifying assumption of infinite population size, and predict little effect of network topology on the fixation probability of a beneficial mutation (Maruyama, 1970; Slatkin, 1981). By contrast, models employing EGT, in which individuals occupy the vertices of a graph and edges represent dispersal routes between neighboring sites, predict that fixation probabilities can change based on how the nodes are connected (Lieberman et al., 2005).

To see this more clearly, it is helpful to consider the predictions of each model in one of the simplest possible scenarios: a four-deme “star” network composed of satellite “leaves” and a central “hub” (Figure 1). Both migration-selection models in population genetics and EGT predict that a rooted -star-network, where one deme supplies more individuals to others than it receives (Figure 1A, left panel), can decrease (suppress) fixation probabilities relative to a well-mixed system (Figure 1A, middle panel). A beneficial mutant is likely to spread through this topology only if it arises, as in the case illustrated, in the hub, and selection is substantially stronger than migration. The predictions made by standard population genetic models and EGT differ for star networks with connections clustered at a few vertices (Figure 1A, right panel). Relative to a well-mixed system, population genetic models predict no effect of topology on the rate or probability of spread, while EGT predicts fixation probabilities can increase, an effect termed amplification, because beneficial mutations arising in a leaf can spread to all other patches via the central hub (Lieberman et al., 2005; Pavlogiannis et al., 2018).

**Figure 1.**
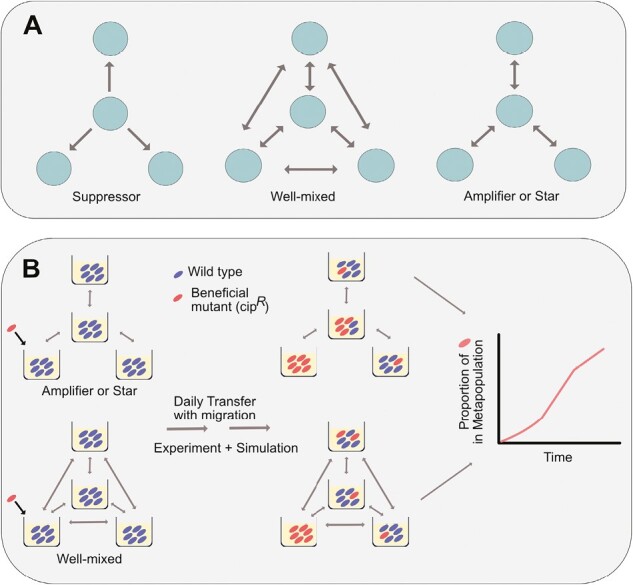
Network Topologies and experimental design: (A) Three network topologies among four subpopulations. Arrows depict dispersal among subpopulations (green circles). (B) Experimental schematics.

The EGT approach has inspired a rich theoretical literature exploring the potential for ever more complicated network structures to serve as amplifiers of selection (Galanis et al., 2017; Pavlogiannis et al., 2018; Tkadlec et al., 2021), including claims that certain topologies, like the so-called “superstar,” could asymptotically amplify even very small fitness differences. Others (Galanis et al., 2017; Jamieson-Lane & Hauert, 2015) tempered these findings by showing that this prediction depends on model details like the ordering of birth and death steps (Hindersin & Traulsen, 2015; Tkadlec et al., 2020) and that perfect amplification would require unlimited space and time (Tkadlec et al., 2021). In any case, the central claims of EGT remain untested by experiment in any biological system, and so its relevance to real-world situations remains uncertain. Moreover, because EGT rests on a stochastic model of evolution in finite populations (Moran, 1962) in which individuals occupy the nodes of the graph, the empirical robustness of its predictions to alternative scenarios where nodes are composed of subpopulations, variable rates of dispersal, or migration asymmetries (Adlam et al., 2015; Constable & McKane, 2014; Houchmandzadeh & Vallade, 2011, 2013; Marrec et al., 2021) remains unclear.

Here, we evaluate the impact of network topology on the fixation dynamics of a beneficial mutation directly through experiment. Specifically, we focus on the spread of an antibiotic-resistant mutant through a star topology across a range of dispersal rates, as this is the simplest network structure where EGT and standard population genetics make divergent predictions. The well-mixed topology serves as a control. Our experiment tracks the spread of an initially rare (1:1000) ciprofloxacin-resistant (cip^R^) mutant of *Pseudomonas aeruginosa* strain 14 (PA14-*gyrA*) invading four-patch metapopulations varying in topology and dispersal rate (Figure 1B). Selection is uniform across all patches and is imposed by supplementing growth media with subinhibitory concentrations of ciprofloxacin adjusted to provide a ~20% fitness advantage to the resistant mutant. Dispersal occurs during daily serial transfer by first mixing samples from the appropriate subpopulations and then diluting the mixture to adjust dispersal rates (see Methods for details). Since the theory makes predictions about the fixation of a single beneficial mutation, we focus on the first 5–6 days (~6.67 generations/day: ~35–40 generations) to minimize the opportunity for de novo mutations rising to high frequency. We keep track of the frequency of the beneficial mutant over time, which should closely approximate the probability of fixation because the larger the frequency of a mutant at time *t*, the more likely it is to eventually fix. As such, we use “amplification” here to mean the increased rate of spread of a beneficial mutation *relative* to that expected from the well-mixed case at a given time, rather than the fixation probability itself. We check our experimental results and the correspondence between rate of spread and probability of fixation, with a new agent-based simulation (see Methods and Supplementary Information) in which an individual is represented by an agent that competes for finite spaces in a node and can disperse along edges. Together, our results allow us to test directly, both in vitro and in silico, whether network topology modulates the fixation process that drives adaptive evolution, and if so, how this occurs.

## Results

**Figure 2.**
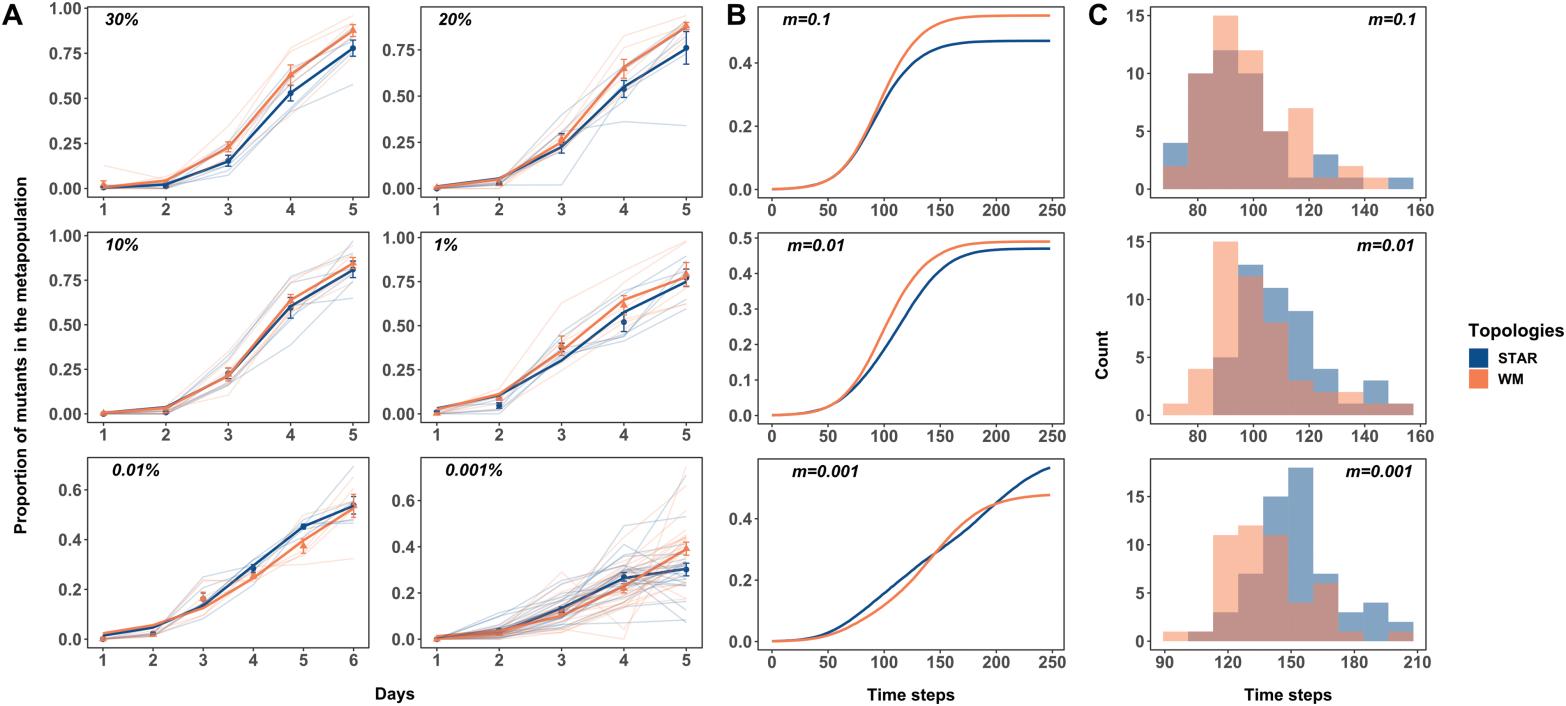
The proportion of cip^R^ mutant in replicate metapopulations propagated by either star (blue) or well-mixed (red) networks with unweighted migration. The bright lines depict the nonlinear least squares (NLS) fit for each metapopulation structure. Panel A shows experimental results; simulation results are shown in (B) and (C). Migration rates are noted in the inset of each plot. Migration rates in the simulation means a low, intermediate, or high value relative to the effective carrying capacity of the patch. Five days in the experiment is equivalent to approximately 5 days or 240 timesteps in the simulation (see methods for details on how time steps in simulations compare to days in experiment). (C) Histograms from the simulations showing the minimum generations required for the cip^R^ to reach a frequency of at least 50% in a metapopulation under each combination of network type and migration rate. Each data point represents the mean proportion of all of the replicate metapopulations on that day, and error bars represent standard error (*SE*).

Our results show that the effect of network topology on the spread of the cip^R^ mutant depends on migration rate (Figure 2). The beneficial mutant spreads faster through a well-mixed than a star topology at migration rates above 10% (Figure 2A; final frequency of cip^R^ in networks with 30% migration: χ^2^ = 15.348, *p* < 0.0001; 20% migration: χ^2^ = 9.0148, *p* = 0.0027), while the rate of spread is statistically indistinguishable at the intermediate migration rates of 10% and 1% (10% migration: χ^2^ = 0.7082, *p* = 0.4001; 1% migration: χ^2^ = 0.2168, *p* = 0.6415). Below migration rates of 0.01% we see evidence that cip^R^ spreads modestly faster in a star network than in a well-mixed system, consistent with the prediction from EGT that bidirectional star networks can amplify selection. Notably, the amplification effect is transient, being maximal at intermediate time steps (0.01%: relative frequency of cip^R^ to WT on Day 5: χ^2^ =13.825, *p* = 0.0002 and 0.001%: frequency of cip^R^ on Day 4: χ^2^ = 5.2577, *p* = 0.0218) and disappearing on Day 5 or 6, depending on the migration rate (see Supplementary Table 3), an effect that has not been previously observed in models of EGT.

Our results suggest that star topologies can increase the rate at which a beneficial mutation spreads, although its effects appear limited to very low migration rates. An alternative explanation is that the increased rate of spread in our experiments was due to the de novo evolution of second-site beneficial mutations in the cip^R^ background. Three lines of evidence argue against this interpretation. First, there should be no inherent evolutionary advantage to any treatment in our experiment because mutation supply rates, being the product of population size and mutation rate, did not differ across treatments. Second, we observed novel colony morphotypes in the more abundant wild-type background only that, when present, had undulate morphologies indicative of biofilm formation. Third, we never recovered mutants more resistant than our focal cip^R^ strain, even after propagating the wild type for 10 days under identical conditions (see Methods, Supplementary Figure 8), suggesting that spontaneous resistant mutations, if present, remained too rare to influence our results. Our observation that the amplification of an initially rare beneficial mutant occurred in spite of potential competition from de novo mutants in the more abundant competitor class thus makes our results even more compelling.

To confirm these results are not an idiosyncratic feature of our biological system, and to provide additional insight into the mechanisms driving amplification, we simulated the population dynamics of selection in metapopulations under the same topologies and migration rates using an agent-based model. The model tracks competition between wild-type and resistant bacteria for a fixed number of spaces in each patch with dispersal along edges between patches, with fitness being given by the probability of being killed by the antibiotic, and population sizes within each patch being allowed to vary between zero and a fixed carrying capacity. Our model thus allows us to capture the dynamics of slow, but nonequilibrium, migration. Simulation (Figure 2B-C) and experimental results match closely, with the well-mixed topology being faster at spreading the beneficial mutant than the star network at high migration rates. As in our experimental results, transient amplification was seen at low migration rates (Figure 2B). Closer inspection reveals amplification is most likely to occur when the expected number of mutant migrants per generation along each edge, at the effective carrying capacity of mutants, is on the order of one. This corresponds to:


$$\begin{array}{l} \begin{array}{lll} & Expected\;migrants=(Migration\;rate)\;\times (Spaces\;per\;node)\; & \times [1-Antibiotic/Resistance]∼1\; \end{array} \end{array}$$

where *Antibiotic* is the antibiotic concentration and *Resistance* represents the reduction in the kill rate of mutants normalized by their growth rate, so that all of them would be killed when (*Antibiotic/Resistance*) ≥ 1. This result suggests that amplification associated with “slow” migration rates is caused by seeding events of mutants into a new subpopulation if and only if the mutants have already successfully colonized a previous subpopulation. Substitution occurs in a more predictable and stepwise fashion under slow, relative to fast, migration rates because beneficial mutants have more time to rise to high frequency in the subpopulation they previously colonized, and so are less likely to be lost due to drift when colonizing a new subpopulation. Moreover, a star network that concentrates incoming migrants into the hub will alleviate genetic drift more than a well-mixed network, and should, in principle, act as a stronger amplifier of selection. By contrast, when migration introduces beneficial mutants at a rate faster than they are lost due to genetic drift, a better-connected well-mixed network spreads beneficial mutants faster than a star network where leaves are only connected via the hub.

Recent theoretical work (Marrec et al., 2021; Yagoobi & Traulsen, 2021) treating nodes as subpopulations rather than individuals, as in our experiments, shows that migration asymmetry between leaves and hub can potentially amplify fixation probabilities relative to the well-mixed case. Specifically, star networks with net outward or inward migration are predicted to be suppressors or amplifiers of selection, respectively, while those with no asymmetry (balanced) migration should have no advantage over a well-mixed network in fixing a beneficial mutant (Marrec et al., 2021). Our first experiment (Figure 2) adjusted the migration rate, *m*, to ensure all patches received the same number of mutants. Using the same experimental setup, we can test these predictions by manipulating the relative amount of migration between the hub and the three leaves of the star network in both experiments and simulations.

**Figure 3.**
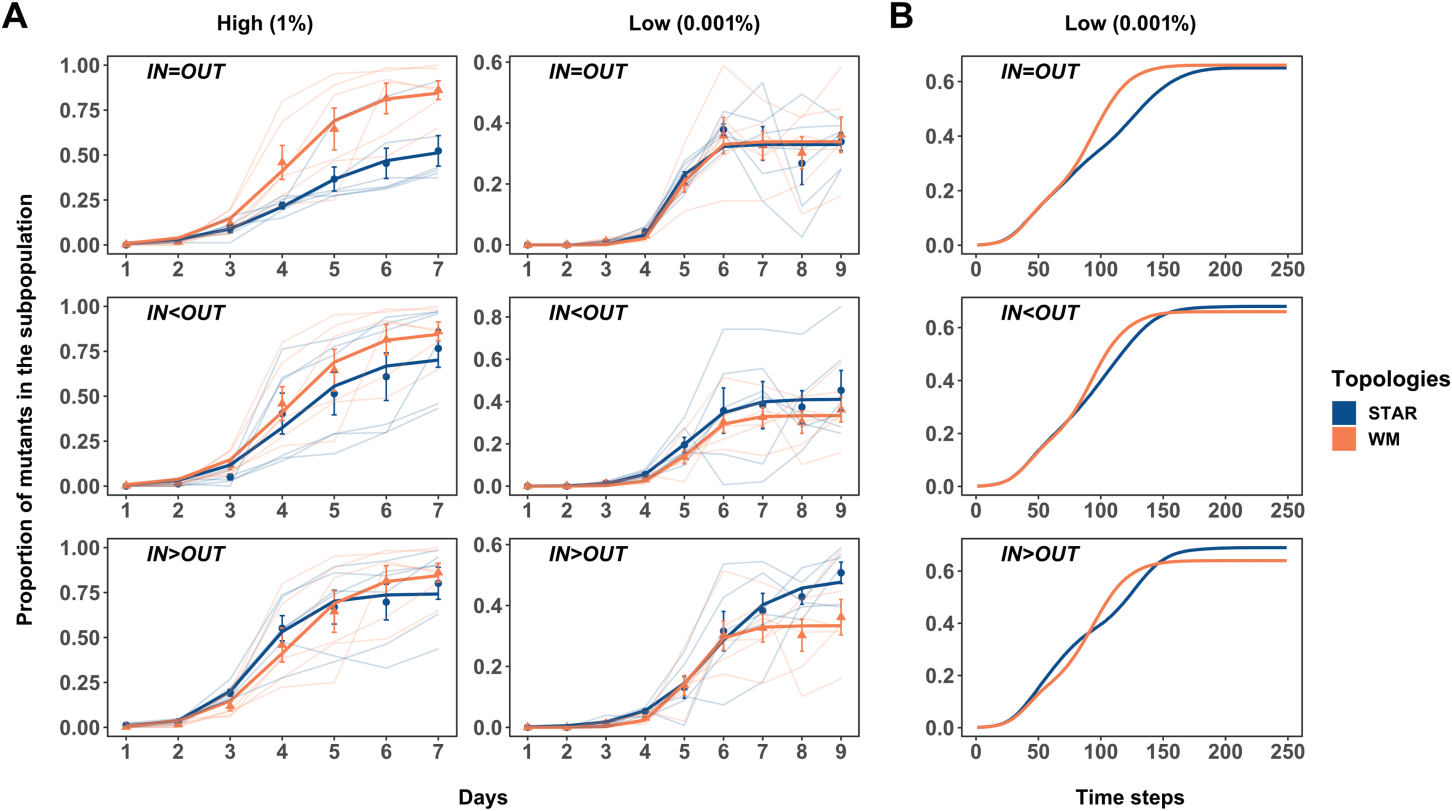
The proportion of cip^R^ mutant in replicate metapopulations propagated on either star (blue) or well-mixed (red) networks with weighted migration. Bright lines are the nonlinear least squares (NLS) fit to the two network treatments. (A) shows results from the experiments under high and low migration rates, whereas (B) shows the results of the agent-based model only under the low migration rate. The respective dispersal asymmetries are provided in the inset of each plot. Each data point represents the mean proportion of all of the replicate metapopulations on that day, and error bars represent standard error (*SE*).

Our results are consistent with the predictions of the theory. At high migration rates (Figure 3A), the rate at which the cip^R^ mutant spreads is never statistically significantly higher than that of the well-mixed topology under both forms of asymmetric migration (middle and bottom panels; χ^2^ = 1.1888, *p* = 0.2756 (OUT>IN) and χ^2^ = 0.4124, *p* = 0.5208 (IN>OUT), respectively; see Supplementary Table 4), and is substantially slower when migration rates were balanced among the nodes (top panel) (IN=OUT: relative frequency of cip^R^ on Day 9: χ^2^ = 12.234, *p* = 0.0005). At low migration rates (Figure 3B), however, the dynamics of cip^R^ spread are indistinguishable (see Supplementary Table 4) from those of the well-mixed case for both balanced migration (top panel, χ^2^ = 0.1848, *p* = 0.6673) and net outward migration (middle panel, χ^2^ = 0.5233, *p* = 0.4694), as expected from theory. When inward migration exceeds outward migration (bottom panel), however, the cip^R^ mutants gain a significant advantage in the latter stages of the experiment (IN>OUT: relative frequency of cip^R^ on Day 9: χ^2^ = 5.2917, *p* = 0.0214). The results of our simulations are shown in the panels in Figure 3B and agree well with our experimental results.

Whether or not amplification occurs should depend on the balance between two dynamic processes—the rate of fixation within a patch and the rate of dispersal to new patches. When migration rates are fast, an initially rare beneficial mutant cannot reach a sufficiently high frequency in its native patch to guarantee dispersal to other patches. Consequently, if it does manage to get dispersed to a novel patch, the beneficial mutant is initially so rare that it is likely to be lost due to drift. Under slow migration, however, selection increases the frequency of a beneficial mutant faster in its native patch than it is dispersed to novel patches, ensuring that it can be repeatedly dispersed to novel patches and so reduce the likelihood of drift loss upon arrival. Amplifying topologies act in a similar way when dispersal is slow, by allowing beneficial mutants to first fix in the patch where they were introduced, and then funneling them through a central hub, so the likelihood of drift loss before other leaves are seeded is reduced. Under well-mixed conditions, migration overwhelms selection, such that the constant influx of lower fitness migrants from other patches means the beneficial mutant cannot accumulate to a sufficiently high frequency in its focal patch before it is dispersed to other patches, where it is rare and likely to be lost due to drift.

**Figure 4.**
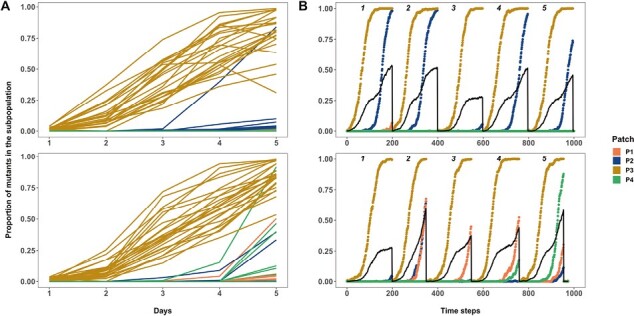
The proportion of the cip^R^ mutants in the constituent subpopulations of each replicate metapopulation propagated as either star (both A and B, first row) or well-mixed (both A and B, second row) networks with unweighted migration of 0.001% (~100 individuals). (A) is the data from the experiment, and (B) is the data from the agent-based model. Subpopulation nomenclature: P3 = node of introduction of the cip^R^ mutant, P2 = hub and P1 and P4 = rest of the peripheral leaves. In (A), cip^R^ fixation dynamics in four subpopulations of each of the 24 replicate metapopulations under each network are shown (top = star, bottom = well-mixed). The black solid lines in (B) are the overall proportion of cip^R^ mutants in a metapopulation. In (B), five replicate instantiations (each run for 200 generations) of the simulation are shown for each network (top = star, bottom = well-mixed).

We evaluated this interpretation by examining the dynamics of the cip^R^ mutant as it spreads among subpopulations in our experiment. Fixation is expected to occur first in the leaf in which the beneficial mutant was initially inoculated followed by, in an amplifying star network, accumulation in the hub and then spread to other leaves of the network. In a well-mixed metapopulation, however, the spread of the cip^R^ mutant should occur in both hub and leaf subpopulations at the same time. Indeed, when we examine the dynamics of the cip^R^ mutant in star and well-mixed metapopulations at a low migration rate (0.001%) where amplification is seen in the former but not the latter, we see the expected patterns (Figure 4). The cip^R^ mutant first fixes in the leaf where it was initially inoculated for both networks, as expected. Although there is substantial variation among replicates, our results show that the cip^R^ mutant spreads differently among the remaining three subpopulations in the two kinds of network: In the star network, there is a clear tendency for the mutant to spread from the initial subpopulation to the hub (P2) first, whereas in the well-mixed network, the mutant is equally likely to spread to the hub as any additional subpopulation (Figure 4A). These experimental results are closely mirrored by those of the simulation (Figure 4B).

**Figure 5.**
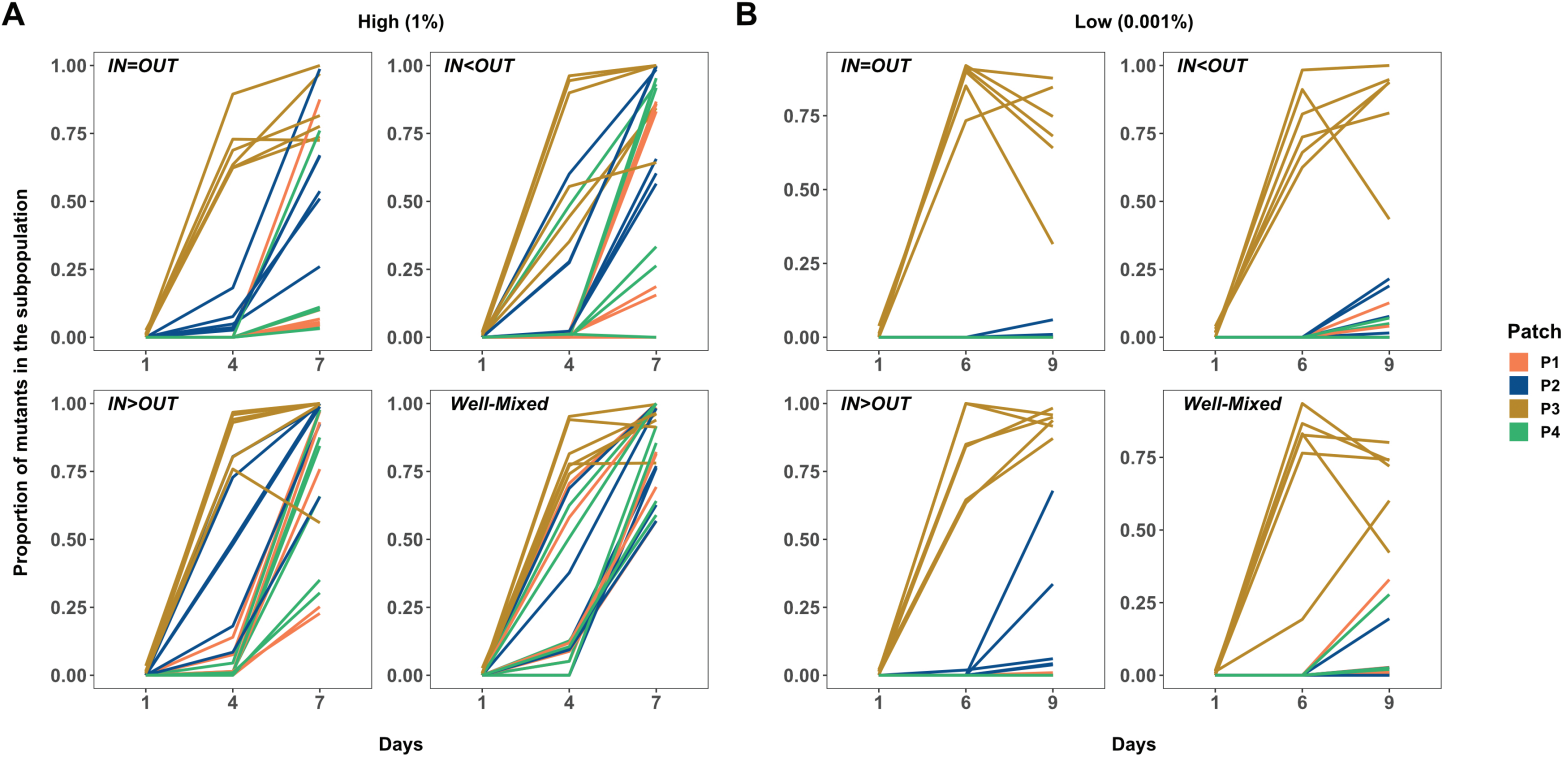
The proportion of the cip^R^ mutants in the constituent subpopulations of each metapopulation propagated by either asymmetric star or well-mixed networks under high (A: 1% or 10^5^ individuals) and low (B: 0.001% or 10^2^ individuals) weighted migration. Subpopulation nomenclature: P3 = node of introduction of the cip^R^ mutant, P2 = hub and P1 and P4 = rest of the peripheral leaves. In both (A) and (B), cip^R^ fixation dynamics in four subpopulations of each of the six replicate metapopulations using each of the three asymmetric star networks and the well-mixed network are shown (see plot insets for details) under high and low migration rates, respectively.

We see similar dynamics of spread among subpopulations in our experiments examining migration asymmetry. In well-mixed systems and those star networks where amplification was not observed (high migration rates), the cip^R^ mutant rapidly spreads into both hub and leaves after near fixation in the patch of introduction (Figure 5A). In contrast, the cip^R^ mutant spreads to the hub first in the most strongly amplifying star topology (IN>OUT), consistent with the idea that beneficial mutants are more likely to avoid stochastic loss due to drift by being concentrated in the hub (Figure 5B).

**Figure 6.**
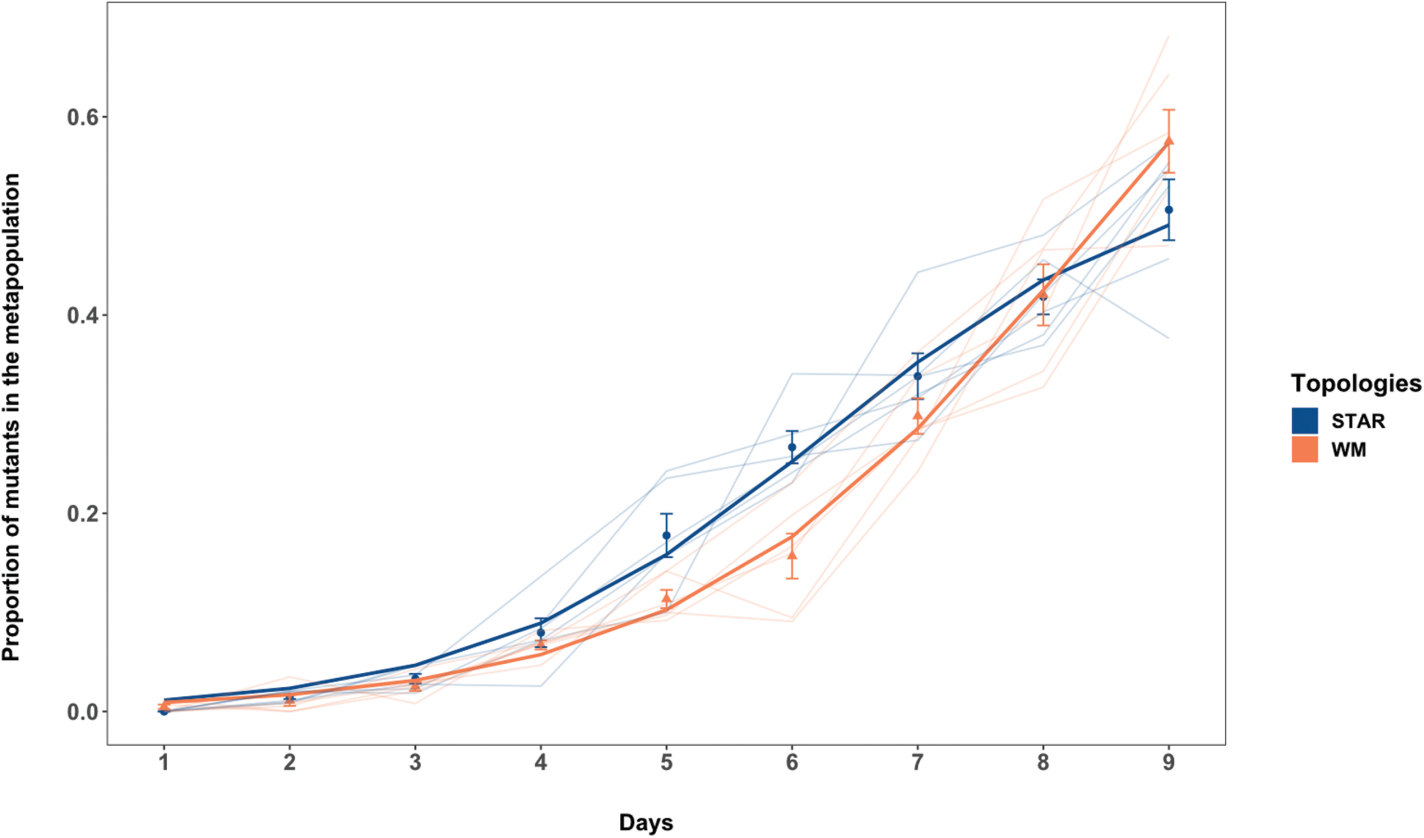
The proportion of cip^R^ mutant in replicate metapopulations propagated on either an inward star (blue) or well-mixed (red) networks with low population size (10^5^ CFU/ml) and low migration rate (10^3^ CFU/ml). Bright lines are the nonlinear least squares (NLS) fit to the two network treatments. Each data point represents the mean proportion of all of the replicate metapopulations on that day, and error bars represent standard error (*SE)*.

If avoiding “drift loss” under low migration rate is indeed the mechanism for amplification, then imposing more severe drift should increase the magnitude and duration of the observed amplification. We tested this hypothesis by enforcing a stricter bottleneck, and thus stronger drift, during daily transfers and tracking the spread of resistant mutants in a star metapopulation with in-weighted migration (IN>OUT) and a well-mixed metapopulation at a low migration rate (~1,000 individuals). The results are consistent with our prediction (Figure 6): For intermediate time points (Days 5 and 6), there was a significantly higher proportion of mutants (Day 5: χ^2^ =17.255, *p* = 3.269e-05, Day 6: χ^2^ =16.17, *p* = 5.79e-05) in the star-like metapopulations compared to the wild type. In other words, we observed an amplification with a higher magnitude and longer duration (stable for ~30 generations, which was nearly the entire length of our previous experiments). This result is consistent with our hypothesis that the likelihood of drift loss is lower in star-like metapopulations when the migration rate between subpopulations is low and asymmetric migration concentrates mutants through the hub. Our results thus lend strong support to the idea that a reduction in the probability of drift loss is responsible for the amplification effect in star-like topologies. Moreover, this result emphasizes the need for future theoretical and experimental work to focus on fine-tuning evolutionary forces such as stochastic drift, selection, and migration to determine the magnitude of amplification in different topologies.

## Discussion

We have shown experimentally and through simulation that metapopulation structure can impact the dynamics of adaptive substitution. Star metapopulations, in which leaf subpopulations exchange migrants through a central hub, can act as amplifiers of selection, leading to faster rates of spread than a comparable well-mixed population where all subpopulations share migrants equally. Amplification is most pronounced when selection is strong relative to migration, a scenario that reduces the probability that a rare beneficial mutant in a newly colonized patch will be lost due to drift, and when the topology of dispersal concentrates the beneficial mutant in a central hub (“inward” > “outward” migration). In other words, amplification occurs because rare mutants are less likely to be lost, not because the strength of selection itself increases.

This result is remarkable because it was not anticipated by standard theory in population genetics, in which population structure usually has little effect on the probability of fixation for beneficial mutants. This conclusion likely derives from the tradition in population genetics of considering allele frequency changes in the limit of infinite populations and high migration rates. Our results, by contrast, show how stochastic effects associated with finite population sizes can alter the dynamics of adaptive substitution in ways that are consistent with predictions from EGT where individuals are assigned to nodes of a graph. Importantly, our results show that amplification can occur under a broader and arguably more realistic set of conditions where populations, not individuals, occupy the nodes. Our work emphasizes the previously overlooked importance of migration rate and serves as a first step toward bridging these two approaches, with infinite populations on the one hand and finite populations focused on the dynamics of individuals on the other.

More generally, it will be useful to expand the analytical framework of EGT to include more biological realism and to articulate more precisely the range of conditions under which amplification can occur. It should be possible, for example, to use network topology to amplify the selection of even a slightly favored mutation for the purpose of experimentation or the directed evolution of desired traits in industrial applications. A more comprehensive theory of evolution on structured landscapes will also be important in other aspects of biology, including the spread of invasive species, pathogens, and the resistance factors they possess.

## Methods

### The SANCTUM model

Each node (*A*_*i*_) has *n*_*i*_ spaces that can each be empty, occupied by a wild type, or occupied by a mutant. For these experiments, all the *n* values are set to 1,600. For each generation of the simulation, there are three phases: (i) Death, (ii) Birth, and (iii) Migration. *Death*: An agent is removed during the death step with a probability that depends on the antibiotic concentration divided by its individual resistance. *Birth*: Each agent has a chance of reproducing an identical agent in an empty space of the same node during the birth step. Similar to the Lotka–Volterra model of population growth, the probability of reproduction increases with the number of empty spaces in the node. *Migration*: There is a probability of migration that varies based on the experimental condition. If an agent is selected to migrate, it randomly moves to an adjacent connected node with a probability proportional to the weight of that edge (w). These definitions are interpreted in the model as follows:


$$\begin{array}{l} Effective\;carrying\;capacity\;with\;antibiotic=(1-Z*A/R)K \end{array}$$

where *Z* is first-order kill rate of the mutants by the antibiotic, *A* is the antibiotic concentration, *R* is growth rate of mutants, and *K* is the total number of spaces per node (the unmodified carrying capacity). Using the effective carrying capacity of mutants limited by the antibiotic:


$$\begin{array}{l} \begin{array}{lllll} & Expected\;migrants\;per\;step\;per\;edge=\; & (Migration\;rate)\times (Ef\;fective\;carrying\;capacity)\; & =(Migration\;rate)\times \left[(1-Z*A/R)\;K\right]\; & =(Migration\;rate)\times (Spaces\;per\;node)\times \;[1-Antibiotic/Normalized\;resistance]\; \end{array} \end{array}$$

Here, *R*/*Z* represents the Normalized Resistance, which reflects the balance between the death and growth rates. This value also sets the threshold for *A*, above which all the mutants would be eliminated. The initial system is randomly seeded with one thousand agents across all nodes, and one of these is selected to be the mutant (not in the hub). Time in the simulation is calculated as below:


$$\begin{array}{l} \begin{array}{lll} & Total\;simulation\;time=(time\;equivalent\;in\;experiment\; & (days)\times 24hr\times 60min)/t\; \end{array} \end{array}$$

where *t* is the doubling time (generation time) for PA14 ~ 30 min. Therefore, 5 days of experiment is equivalent to 240 time steps in the simulation. For each simulated condition, the metapopulation fraction is averaged over 100 instantiations. For runs that ultimately fix, the number of generations until the mutants are the majority of agents is also recorded.

### Microbial strains and conditions

For all experiments, clonal populations of *Pseudomonas aeruginosa* strain 14:gyrA (PA14:*gyrA*) and PA14:*lacZ*, isogenic to PA14 except with a point mutation in the *gyrA* gene and an insertion in the *lacZ* gene, respectively, were used. Colonies possessing the *lacZ* insertion appear blue when cultured on agar plates supplemented with 40 mg/l of 5-bromo-4-chloro-3-indolyl-beta-d-galactopyranoside (X-Gal), and are visually distinct from the PA14:*gyrA* white coloration. The neutrality of the *lacZ* marker was confirmed in our experimental environments by measuring the fitness of the marked strain relative to the unmarked strain. Populations were cultured in 24-well plates with 1.5 ml of media in each well, in an orbital shaker (150 RPM) at 37 °C. The culture media consisted of Luria Bertani broth (LB: bacto-tryptone 10 g/l, yeast extract 5 g/l, NaCl 10 g/l) supplemented with 20 ng/ml of the fluoroquinolone antibiotic, ciprofloxacin. This concentration of ciprofloxacin confers ~20% selective advantage to PA14:*gyrA* relative to PA14:*lacZ* (Supplementary Figure 7). All strains and evolving populations were frozen at −80 °C in 20% (v/v) glycerol.

### Evolution experiment

A single metapopulation consisted of four subpopulations, one subpopulation being located on each of four different 24-well plates. Plate 2 was always assigned as the hub, and plates 1, 3, and 4 were treated as the leaves. This design allows us to track up to 24 replicate populations using just four multiwell plates. The experiment was initiated by inoculating each subpopulation with ~10^7^ colony forming units/ml of PA14:*lacZ* descended from a single colony picked from an agar plate and grown overnight in liquid LB at 37°C with vigorous shaking (150 RPM). The cip^R^ mutant, derived from frozen cultures in the same way, was introduced simultaneously into one subpopulation (plate 3) at a density ~10^4^ PA14:*gyrA* cells producing an initial ratio of resistance to wild-type cells of ~1:1000 in this subpopulation. Metapopulations were transferred daily following dispersal among subpopulations (see below) by taking an aliquot corresponding to ~10^7^ CFUs per ml and inoculating into fresh medium. The population density in each subpopulation reached ~10^9^ CFUs, so this transfer regime corresponds to ~6.67 daily generations of growth.

We constructed distinct network topologies by mixing subpopulations prior to serial transfer following the schematic shown in Supplementary Figure 4. Briefly, well-mixed networks were created by combining equal volume aliquots from all subpopulations into a common dispersal pool, diluting this mixture to the appropriate density to achieve the desired migration rate, and then mixing the dispersal pool with aliquots from each subpopulation (so-called “self-inoculation”) before transfer. Star networks, which involve bidirectional dispersal between the hub and leaves, were constructed in a similar way to the well-mixed situation, only now the dispersal pool consisted of aliquots from just the leaves and aliquots from the hub (plate 2) were mixed with “self-inoculation” samples from each leaf prior to serial transfer. Further details on how each network topology and migration rate were achieved are provided in the supplementary material.

### Tracking the spread of resistance

We tracked the spread of the cipR mutant (PA14:*gyrA*) relative to the wild type (PA14:*lacZ*) by plating samples from each subpopulation as well as a mixture of the entire metapopulation on LB agar plates supplemented with X-gal allowing us to use blue-white screening to track the relative frequency of each type over time.

### Statistical analyses

All statistical analyses were conducted using R statistical software (R Core Team, 2020). We used two complementary approaches to analyze our experimental data.

The first models the spread of resistance (see Figures 2 and 3) as a three-parameter logistic growth model using nonlinear least squares with a fixed *N*_0_ (NLS) (Nash, 2016). This model, as with comparable approaches focused on population growth in resource-limited environments, allows us to estimate the rate at which resistance spreads (equivalent to r_max_ in logistic growth) and the final frequency of cip^R^ mutants at the end of the experiment (equivalent to the carrying capacity, K, from logistic growth models) in each replicate metapopulation. Contrasts of maximal growth rates between treatments (star or well-mixed) were performed using a linear model (lm function from base R). Comparable contrasts for the maximum proportion of resistant mutants fixed on the final day of the experiment used a generalized linear mixed model (GLMM) using methods as described below.

The second approach modeled the change in proportion for the cip^R^ mutants directly using a GLMM with quasibinomial error distribution (and logit link function), using the glmmPQL function from the MASS package in R (Venables & Ripley, 2002). We focus on the main effects of time and network structure (star vs. well-mixed) and their interactions at each migration rate treatment. Logistical constraints prevented us from conducting experiments that manipulate both network structure and migration rate simultaneously, so we elected to run separate experiments at each migration rate to focus on the effect of contrasting network structures, as this is the focus of EGT. Our model treats “network” as a fixed effect and “replicate” as a random effect, while accounting for repeated measures through time. This approach produces estimates of the pairwise difference between the slopes (vs. time) for the network treatment (e.g., - Time:Network star - Time:Network well-mixed) that were further analyzed using the EMTRENDS function from the EMMEANS package (analogous to a Tukey post hoc test) (Lenth, 2020). These contrasts allow us to determine the magnitude and direction of the difference between the star and well-mixed networks for the whole experiment.

The approaches above, which focus on estimating the best-fit main effects and interactions, are useful for helping to visualize the dynamics of spread across many instantiations of an inherently noisy process. We additionally focus our attention on contrasts between the fraction of cip^R^ mutants between star and well-mixed treatments at specific days (a) when the fitted logistic model for the star was higher than that of the well-mixed over the course of the complete experiment or (b) when the fitted models reveal a transient “crossover” event at intermediate time steps. We used a GLMM as described above to contrast the fraction of cip^R^ mutants in star versus well-mixed networks at a particular day, treating replicate as a random factor. The analysis of variance of the GLMMs was performed with the ANOVA function from the CAR package.

Full_model <- glmmPQL(Proportion~Time*Treatment, random = 1|Replicate, family = quasibinomial, data)

em1< - emtrends(Full_model, pairwise ~ Treatment, var = “Time”)

model_K < - glmmPQL(Proportion ~ Treatment, random= ~1|Rep, family = quasibinomial, data)

model_R< - lm(R~Treatment, data)

## Supplementary Material

qrad047_suppl_Supplementary_Material

## Data code and availability

All raw data, simulation code, and R script used to make the figures are deposited in the Dryad Digital Repository: https://doi.org/10.5061/dryad.5mkkwh7cd (Chakraborty et al., 2023).
