# Supplementary material for: Experimental evidence that network topology can accelerate the spread of beneficial mutations
Source: Evol Lett. 2023 Oct 11;7(6):447–56. doi: 10.1093/evlett/qrad047 (PMC10693003; doi:10.1093/evlett/qrad047)
Supplement: qrad047_suppl_Supplementary_Material [file qrad047_suppl_supplementary_material.pdf]

Supplementary material for "Experimental evidence that network topology can accelerate the spread of beneficial mutations"

Partha Pratim Chakraborty, Louis R. Nemzer, and Rees Kassen

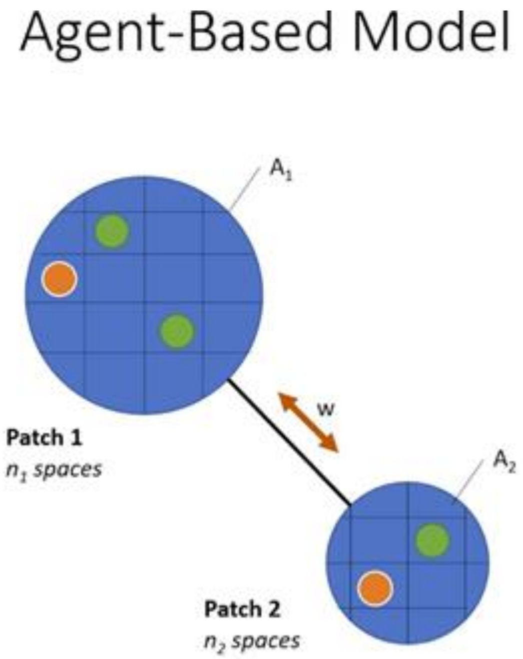

Supplementary Figure 1: Schematic depiction of the algorithm used in the SANCTUM model. See methods section for details.

Modified Agent Based Model with daily bottleneck and sensitive-specific killing:

The original SANCTUM model incorporates selection as antibiotic dependent killing of either the wild type or the mutant. This approach means the uncertainty in selection was introduced in the antibiotic mediated killing step. However, in our experimental model, antibiotics can only kill the sensitive wild-type, not the resistant mutant. To account for this fact and to adhere to the experimental methods better, we modified the original SANCTUM model as described below for a replicate run:

For each Day -

1. The resistant mutant is inoculated in one of the patches in a 1:1000 ratio. All the other patches are inoculated with 1000 wild type agents.
2. Only the wild-type bacteria is killed in the antibiotic mediated killing step according to the antibiotic concentration.
3. Agents grow until they reach the carrying capacity of each patch (in a density dependent fashion that is proportional to the empty spaces remaining at each step).
4. A drift / bottleneck event happens where only 15% of a fully grown patch is carried forward to the next day again for growth.
5. Migration in between the patches happens according to the simulated migration rate and network.
6. Steps 1-5 repeats until the simulation reaches the time limit.

In the modified model, all the parameters remain the same as in the original model except for the introduction of the drift (bottleneck) step. 100 replicates were run for each of the network (STAR or WM) and the frequency of resistant mutants in the metapopulation were recorded at each time step. The simulations were coded in Python.

The results are shown in Supplementary Figure 2 and 3 for the unweighted and weighted migration, respectively. Briefly, and in tune with the original model, we find a close correspondence between the dynamics of spread of the resistant mutant between our experiments and the modified model. Specifically, the modified model closely mirrors the distinct dynamics of spread of resistant mutants in the two topologies, showing a clear advantage to the star topology over the well-mixed topology at the lowest migration rates. Moreover, the dynamics of spread in the modified model also closely matches those observed for the probability of fixation in the original model (compare figures 2b and 3b in the main text with Supplementary figures 2b and 3b, respectively). This last result reassures us that the dynamics of spread is a good proxy for the probability of fixation.

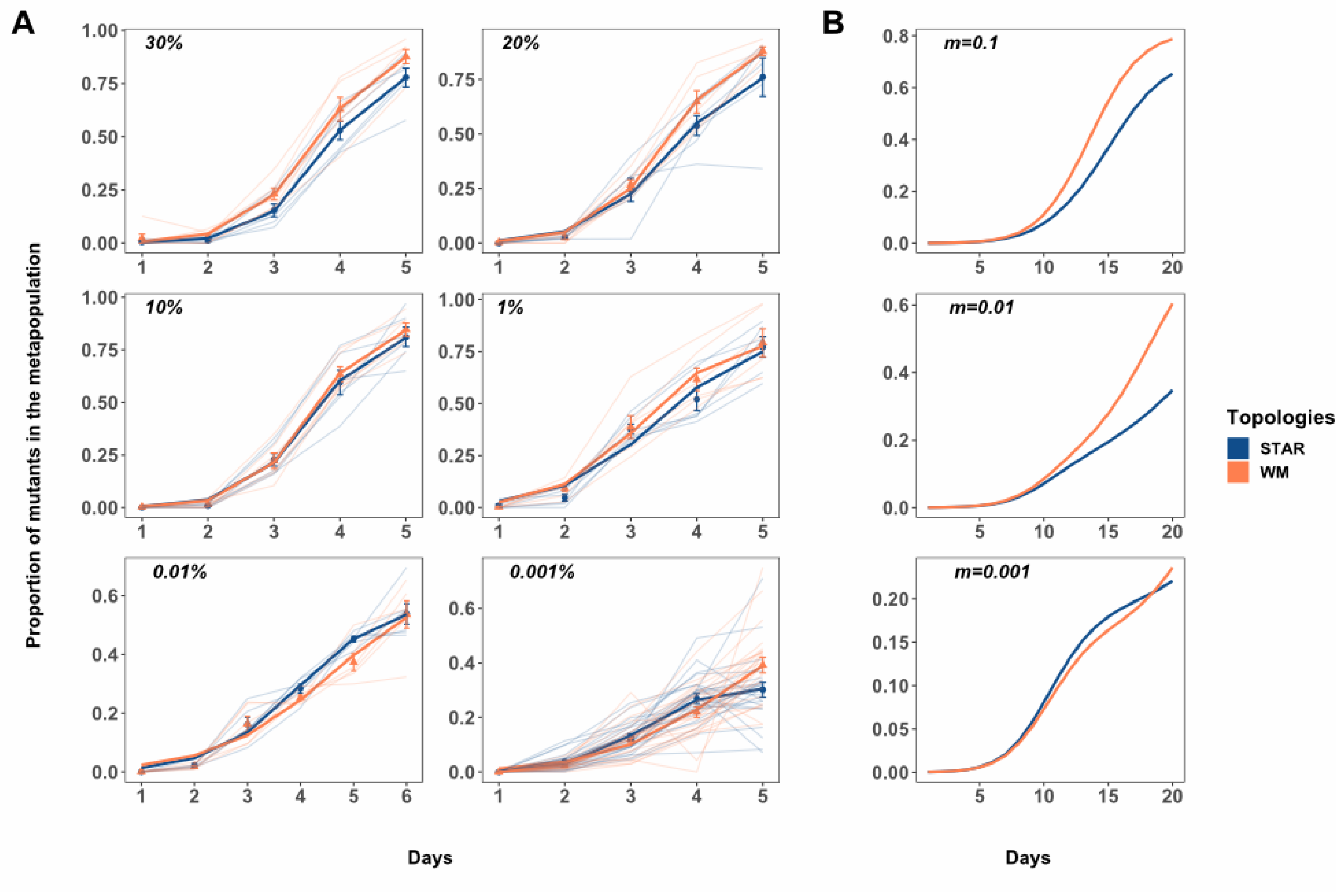

Supplementary Figure 2 : The proportion of cip^R mutant in replicate metapopulations propagated by either star (blue) or well-mixed (red) networks with unweighted migration. Panel A shows experimental results (Fig 2: main text); simulation results (modified) are shown in Panel B. Migration rates are noted in the inset of each plot.

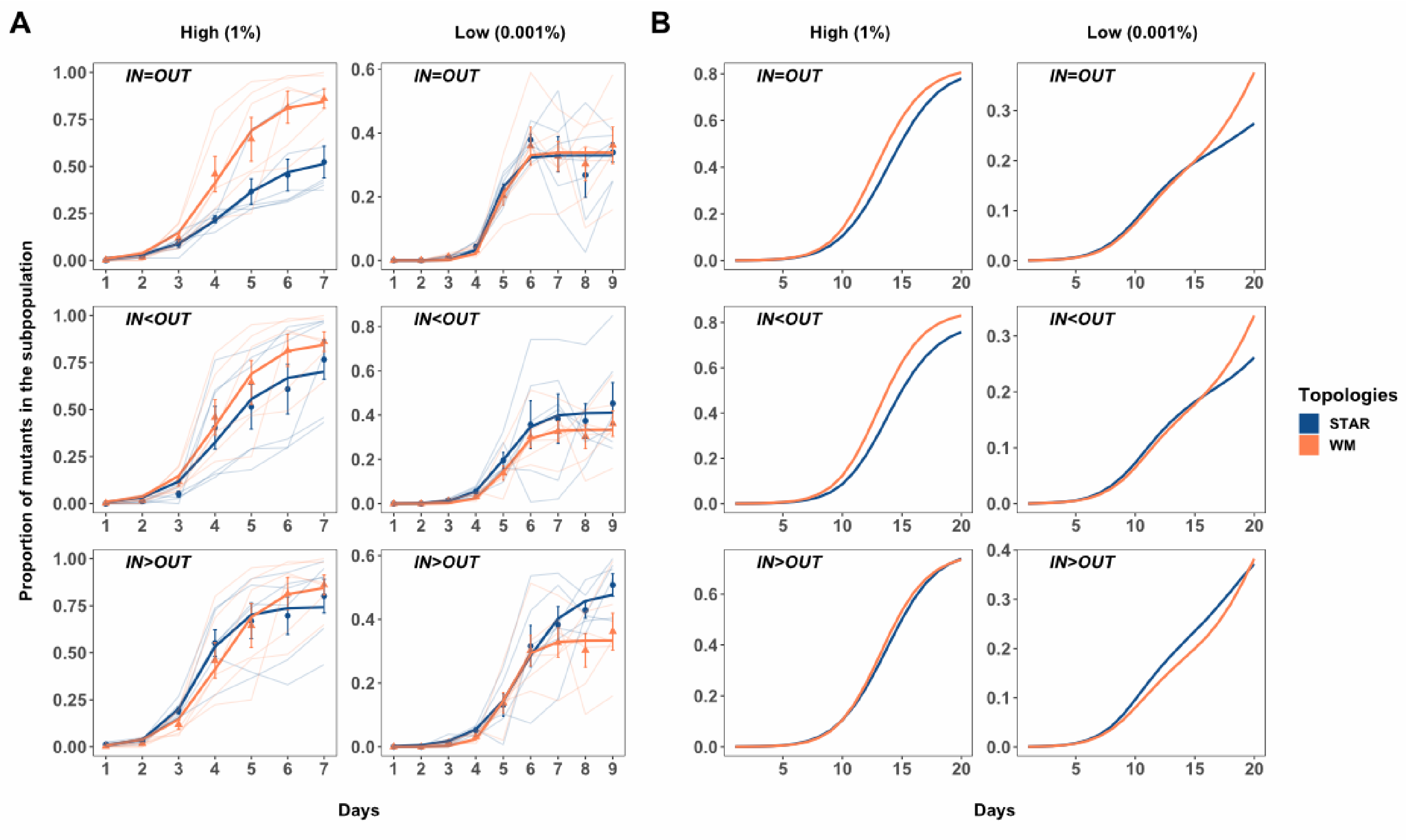

Supplementary Figure 3 : The proportion of cip^R mutant in replicate metapopulations propagated by either star (blue) or well-mixed (red) networks with weighted migration. Panel A shows experimental results (Fig 3: main text); simulation results (modified) are shown in Panel B. Migration rates are noted in the inset of each plot.

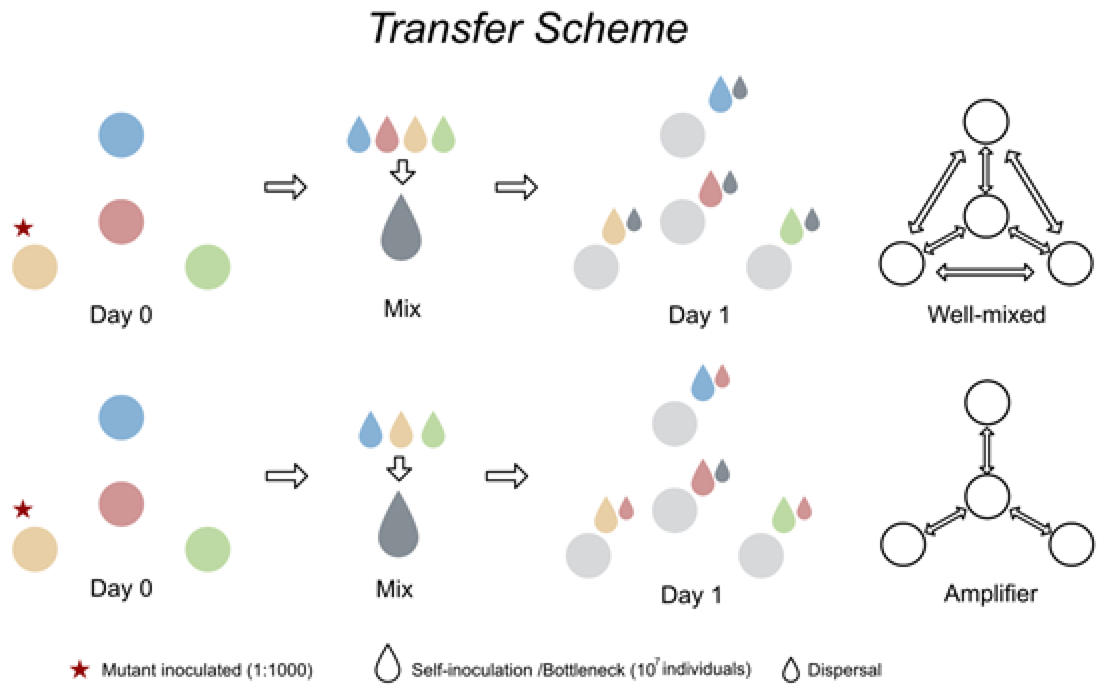

Supplementary figure 4: Transfer scheme to experimentally create star and well-mixed network structures. The red star indicates the patch (P3) where the mutant (PA14-*gyrA*) was inoculated 1:1000 ratio to the wild type (PA14-*LacZ*). Big droplets of the four colors indicate self-inoculation from respective patches whereas big and small gray droplets indicate dispersal mix and dispersal volume, respectively.

Detailed methods used to construct network topologies:

Unweighted migration:

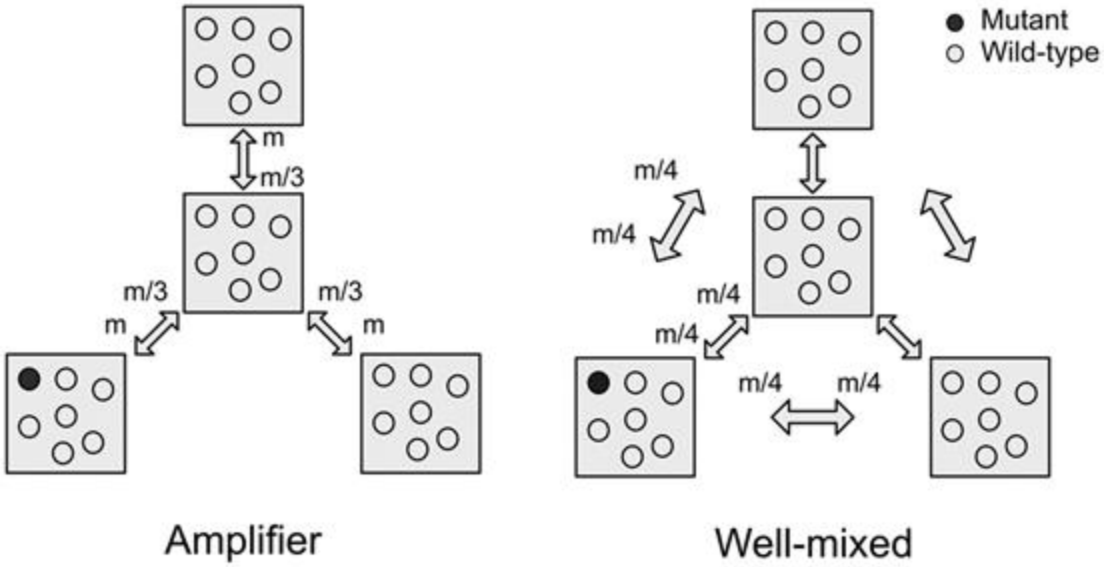

Supplementary Figure 5: Schematics depicting the number of transferred migrants per edge connection in the cases of unweighted migration regime for star/amplifier and well-mixed networks. The value of *m* shows the total number of migrants for each migration treatment, and the fractions are calculated as the contributions from each patch. Filled circles (black) indicate the mutants (PA14-*gyrA*) while initializing the experiment and open circles (clear) are the predominant wild type (PA14-*LacZ*).

Well-mixed: 35 µL of subpopulations 1, 2, 3, and 4 were mixed together, and 20 µL from this resulting pool of migrants (MIX) was serially diluted in fresh media supplemented with 20 ng/mL Ciprofloxacin to achieve ~10^8, ~10^7, ~10^5 and ~10^4 CFU/mL. Then, 15 µL of the diluted MIX was added to 1.5 mL of fresh media along with 15 µL of the previous day's culture to achieve desired migration levels of ~10^6, ~10^5, ~10^3 and ~10^2 CFU/mL. For 20% and 30% migration, 30 µL and 45 µL of the diluted MIX were added with the culture from the previous day.

Star/ Amplifier: 35 µL of subpopulations 1, 3, and 4 were mixed together, and 20 µL from this resulting pool of migrants (MIX) was serially diluted in fresh media and 20 ng/mL antibiotic to reach ~10^8, ~10^7,

~10^5 and ~10^4 CFU/mL. Also, 20 μL of subpopulation 2 (HUB) was serially diluted in fresh media and 20 ng/mL Ciprofloxacin to reach ~10^8, ~10^7, ~10^5 and ~10^4 CFU/mL. Then, 15 μL of the diluted MIX was added to 1.5 mL fresh media along with 15 μL of the previous day's subpopulation 2 culture to achieve the desired migration levels of ~10^6, ~10^5, ~10^3 and ~10^2 CFU/mL. Also, 15 μL of the diluted HUB was added to 1.5 mL fresh media along with the previous day's culture to achieve desired migration levels of ~10^6, ~10^5, ~10^3 and ~10^2 CFU/mL in subpopulations 1, 3, and 4. For 20% and 30% migration, 30 μL and 45 μL of the diluted MIX were added after the bottleneck ("self-inoculation").

Asymmetric migration:

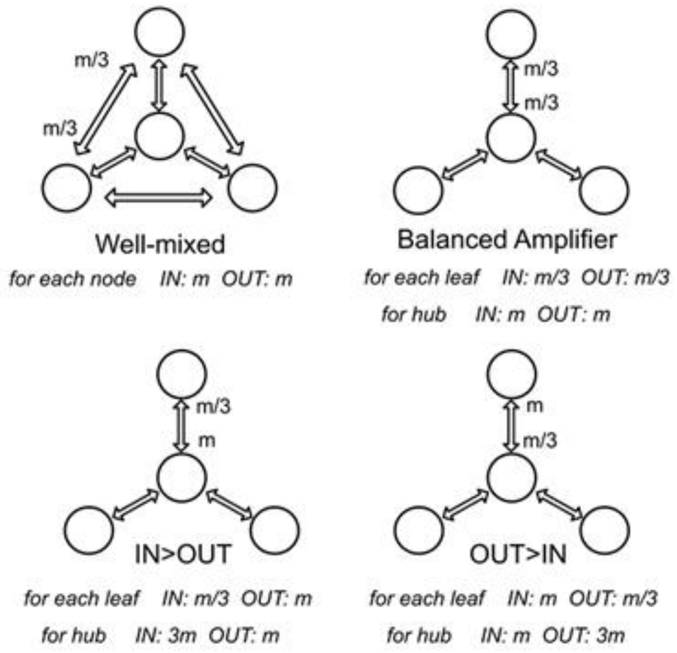

Supplementary Figure 6: Schematics depicting the transferred migrants per edge connection in the case of the weighted migration regimes for three asymmetric star/amplifier networks and the well-mixed network. Each double-sided arrow indicates the number of migrants received and contributed by each patch.

Well-Mixed: 20 µL of subpopulations 1, 2, 3, and 4 (MIX) were mixed in 120 µL of fresh culture media with 20 ng/mL Ciprofloxacin and serially diluted to reach ~4×10^7 or ~4×10^4 CFU/mL. Then, 15 µL of the diluted MIX was added to 1.5 mL fresh media along with 15 µL of the previous day's culture. This resulted in the transfer of 4×10^5 for the high migration rate experiments, or 4×10^2 CFU/mL for the low migration rate.

Balanced star (IN = OUT): 20 µL of subpopulations 1, 3, and 4 (MIX) were mixed in 140 µL of fresh culture media with 20 ng/mL Ciprofloxacin and serially diluted to reach ~3×10^7 or ~3×10^4 CFU/mL. Also, 20 µL of subpopulation 2 (HUB) was mixed in 180 µL of fresh culture media with 20 ng/mL Ciprofloxacin and serially diluted to reach ~10^7 or ~10^4 CFU/mL. This resulted in the transfer of ~3×10^5 and ~3×10^2 CFU/mL to the hub, and ~10^5 and ~10^2 CFU/mL to the peripheral leaves, for the high and low migration rates, respectively.

OUT>IN regime: 20 µL of subpopulations 1, 3, and 4 (MIX) were mixed in 140 µL of fresh culture media with 20 ng/mL Ciprofloxacin and serially diluted to reach ~3×10^7 or ~3×10^4 CFU/mL. Also, 60 µL of subpopulation 2 (HUB) were mixed in 140 µL in fresh culture media with 20 ng/mL Ciprofloxacin and serially diluted to reach ~3×10^7 or ~3×10^4 CFU/mL. This resulted in the transfer of ~3×10^5 or ~3×10^2 CFU/mL to the hub and ~3×10^5 or ~3×10^2 CFU/mL to the peripheral leaves, for the high and low migration rates, respectively.

IN>OUT regime: 60 µL of subpopulation 1, 3, and 4 (MIX) were mixed in 20 µL of fresh culture media with 20 ng/mL Ciprofloxacin and serially diluted to reach ~9×10^7 or ~9×10^4 CFU/mL. Also, 20µL of subpopulation 2 (HUB) was mixed in 180 µL of fresh culture media with 20 ng/mL Ciprofloxacin and serially diluted to reach ~10^7 or ~10^4 CFU/mL. This resulted in the transfer of ~9×10^5 or ~9×10^2 CFU/mL to the hub and ~10^5 or ~10^2 CFU/mL to the peripheral leaves, for the high and low migration rates, respectively.

Low population size:

Well-mixed: 20 µL of subpopulations 1, 2, 3, and 4 (MIX) were mixed in 120 µL of fresh culture media with 20 ng/mL Ciprofloxacin and serially diluted to reach ~4 x 10^5 CFU/mL. Then, 15 µL of the diluted MIX was added to 1.5 mL fresh media along with 15 µL of 1:100 diluted the previous day's culture. This resulted in the transfer of ~4 x 10^3 CFU/mL for the migrant and ~10^5 CFU/mL residents ("self-inoculation").

IN>OUT Star: 60 µL of subpopulations 1, 3, and 4 (MIX) were mixed in 20 µL of fresh culture media with 20 ng/mL Ciprofloxacin and serially diluted to reach ~9 x 10^5 CFU/mL. Also, 20 µL of subpopulation 2 (HUB) was mixed in 180 µL of fresh culture media with 20 ng/mL Ciprofloxacin and serially diluted to reach ~10^5 CFU/mL. This resulted in the transfer of ~9 x 10^3 CFU/mL to the hub and ~10^3 CFU/mL to the peripheral leaves, respectively. Every subpopulation also received 15 µL of 1:100 diluted the previous day's culture from itself which resulted in the transfer of ~10^5 CFU/mL residents ("self-inoculation").

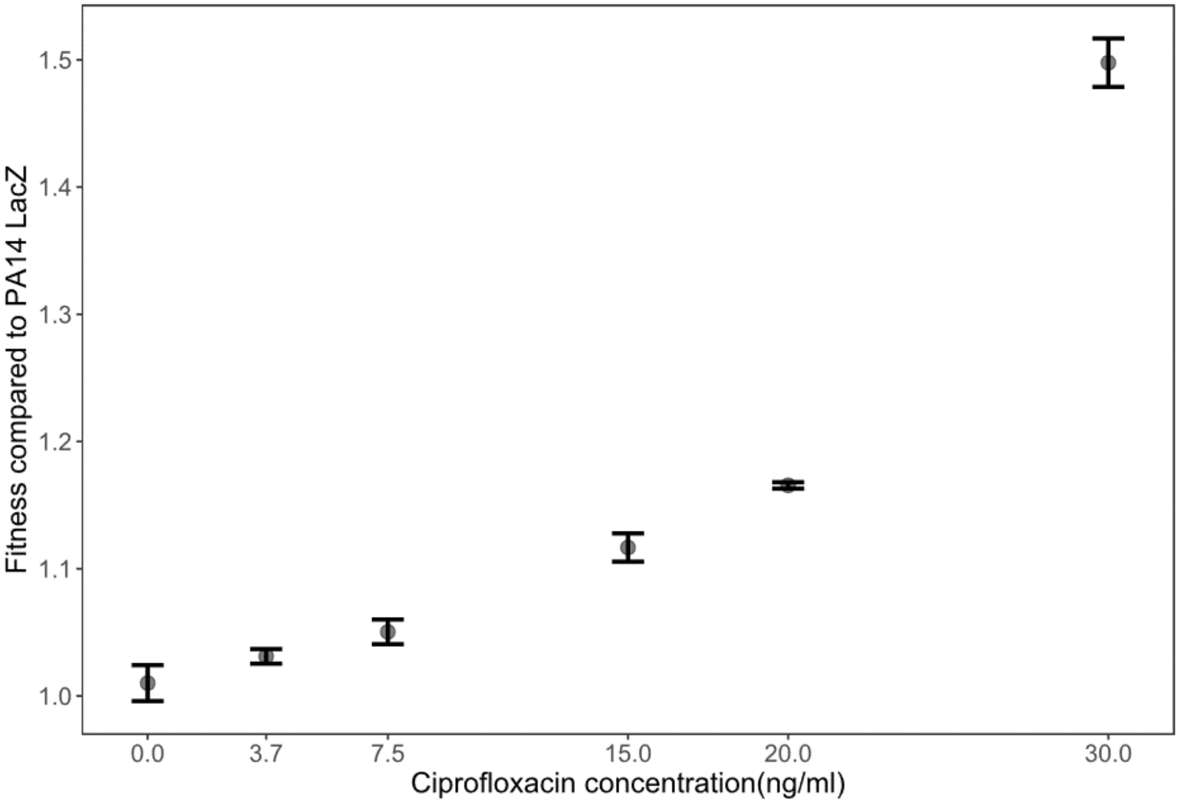

Supplementary Figure 7: Competitive advantage of *PA14-gyrA* in head-to-head competitions (~ 50:50) with *PA14-LacZ* at different sub-inhibitory Ciprofloxacin concentrations. Relative fitness of three biological replicate competitions are shown.

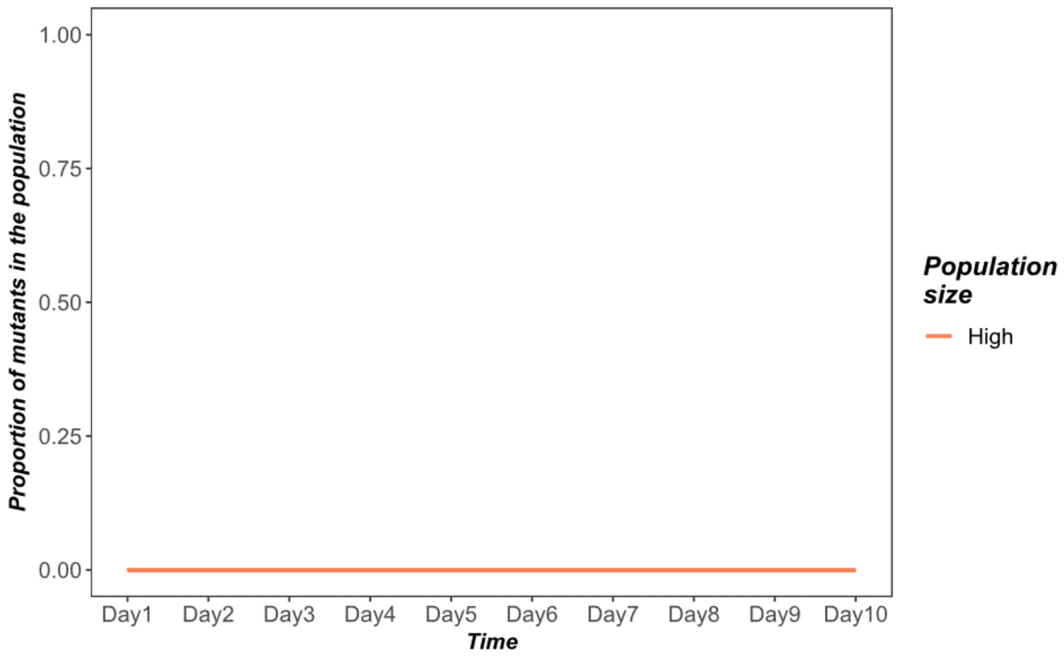

Supplementary Figure 8: Frequency of spontaneous ciprofloxacin resistant (≥ 1μg/ml) mutants derived from the sensitive wild type strain, PA14-*LacZ*, over 10 days of experimental evolution in 20ng/ml Ciprofloxacin. A proportion of zero means there were no detectable colonies capable of growing above the MIC of the cip^R strain *PA14-gyrA* (T83I) used in our experiments.

Statistical results for the full models and the nls:

Migration rate	Simple slope contrasts (Trt1 - Trt2)*time	Estimate	SE	DF	t ratio	p-value
**30%**	STAR - WM	-0.0212	0.156	51	-0.136	0.8923
**20%**	STAR - WM	-0.32	0.158	51	-2.023	0.0483
**10%**	STAR - WM	-0.0915	0.156	51	-0.585	0.5613
**1%**	STAR - WM	-0.0158	0.139	51	-0.114	0.9097
**0.01%**	STAR - WM	0.011	0.0834	63	0.132	0.8956
**0.001%**	STAR - WM	-0.198	0.0732	213	-2.708	0.0073

Supplementary table 1: Statistical analyses of full models for experiments with unweighted migration. For each statistical analysis, the estimated analyzed slope contrast for the GLMM, standard error (SE), degrees of freedom, t-ratio, and p value are shown.

Migration rate	Simple slope contrasts (STAR x - WM)* Time	Estimate	SE	DF	t ratio	p-value
**High**	In = Out	-0.489	0.121	140	-4.032	0.0005
	In < Out	-0.111	0.134	140	-0.832	0.8392
	In > Out	-0.295	0.121	140	-2.432	0.0758
**Low**	In = Out	-0.0364	0.0743	188	-0.490	0.9612

	In < Out	0.0695	0.0766	188	0.907	0.8012
	In > Out	0.1376	0.0797	188	1.728	0.3123

Supplementary table 2: Statistical analyses of full models for experiments with weighted (asymmetric) migration. For each statistical analysis, the estimated analyzed slope contrast for the GLMM, standard error (SE), degrees of freedom, t-ratio, and p value are shown.

Unweighted migration:

Migration rate	Rate of increase (analogous to R in a logistic growth model)	Final frequency (analogous to K in a logistic growth model)	Conclusion:
30%	$F_{1,10}$ =1.623, p = 0.2315	χ^2 = 15.348, p = 8.94e-05 (EXPT 5th day)<br>χ^2 = 8.4269, p = 0.003697 (NLS)	WM has a significantly higher K but R is not significantly different between STAR and WM
20%	$F_{1,10}$ = 0.9149, p = 0.3614	χ^2 = 9.0148, p = 0.002678 (EXPT 5th day)<br>χ^2 = 6.5309 , p = 0.0106 (NLS)	WM has a significantly higher K but R is not significantly different between STAR and WM
10%	$F_{1,10}$ = 0.0515, p = 0.825	χ^2 = 0.7082, p = 0.4001 (EXPT 5th day)<br>χ^2 = 0.1777, p = 0.6734 (NLS)	R and K is not significantly different between STAR and WM
1%	$F_{1,10}$ = 0.0258, p = 0.8757	χ^2 = 0.2168, p = 0.6415 (EXPT 5th day)<br>χ^2 = 1e-04, p = 0.9908 (NLS)	R and K is not significantly different between STAR and WM
0.01%	$F_{1,10}$ = 0.1229, p = 0.7331	χ^2 = 0.0021, p = 0.963 (EXPT 6th day)<br>χ^2 = 1.5471, p = 0.2136 (NLS)	R and K is not significantly different between STAR and WM
0.001%	$F_{1,46}$ = 1.542, p = 0.2206	χ^2 = 5.4046, p = 0.02008 (EXPT 5th day)<br>χ^2 = 9.2005, p= 0.00242 (NLS)	WM has a significantly higher K but R is not significantly different between STAR and WM

Supplementary table 3: Statistical analyses of 3 parameter logistic growth model with nonlinear least squares (nls) for experiments with large population size and unweighted (and comparable well-mixed) migration. For each statistical analysis, rate of increase (r) and final frequency of the beneficial mutant (K) are shown. In the case of K, results from both the final day of experimental data and nls models are compared.

Weighted migration:

Comparisons	Migration	Rate of increase (analogous to R in a logistic growth model)	Final frequency (analogous to K in a logistic growth model)	Conclusion
In = Out<br>vs.<br>WM	High (1%)	$F_{1,10} = 4.8754$, $p = 0.05173$	$\chi2 = 12.234$, $p = 0.0004694$ (EXPT 7th day)<br>$\chi2 = 13.19$, $p = 0.0002815$ (NLS)	WM has a significantly higher K but R is not significantly different between STAR and WM
	Low (0.001%)	$F_{1,10} = 0.6108$, $p = 0.4526$	$\chi2 = 0.1848$, $p = 0.6673$ (EXPT 9th day)<br>$\chi2 = 0.8844$, $p = 0.347$ (NLS)	R and K is not significantly different between STAR and WM
Out>In<br>vs.<br>WM	High(1%)	$F_{1,10} = 0.1022$, $p = 0.7557$	$\chi2 = 1.1888$, $p = 0.2756$ (EXPT 7th day)<br>$\chi2 = 0.6612$, $p = 0.4161$ (NLS)	R and K is not significantly different between STAR and WM
	Low(0.001%)	$F_{1,10} = 0.2279$, $p = 0.6434$	$\chi2 = 0.5233$, $p = 0.4694$ (EXPT 9th day)<br>$\chi2 = 2.1761$, $p = 0.1402$ (NLS)	R and K is not significantly different between STAR and WM
In>Out<br>vs.<br>WM	High (1%)	$F_{1,10} = 0.7773$, $p = 0.3986$	$\chi2 = 0.4124$, $p = 0.5208$ (EXPT 7th day)<br>$\chi2 = 1.1907$, $p = 0.2752$ (NLS)	R and K is not significantly different between STAR and WM
	Low(0.001%)	$F_{1,10} = 0.9051$, $p = 0.3638$	$\chi2 = 5.2917$, $p = 0.02143$ (EXPT 9th day)<br>$\chi2 = 5.7524$, $p = 0.01647$ (NLS)	STAR has a significantly higher K but R is not significantly different between STAR and WM

Supplementary table 4: Statistical analyses of 3 parameter logistic growth model with nonlinear least squares (nls) for experiments with large population size and weighted (asymmetric and comparable well-mixed) migration. For each statistical analysis, rate of increase (r) and final frequency of the beneficial mutant (K) are shown. In the case of K, results from both the final day of experimental data and nls models are compared.

Comparisons	Migration	Rate of increase (analogous to R in a logistic growth model)	Final frequency (analogous to K in a logistic growth model)	Conclusion
In>Out vs. WM<br><br>Small population size	1000 cfu/ml	$F_{1,10}$ =0.10674, p = 0.4059	$\chi2$ = 3.562, p = 0.05912 (EXPT 9th day)<br>$\chi2$ = 5.2773 , p = 0.02161 (NLS)	R is not significantly different between STAR and WM.<br><br>K is significantly higher in the NLS fit model but not significantly different from WM for the experimental data from the final day

Supplementary table 5: Statistical analyses of 3 parameter logistic growth model with nonlinear least squares (nls) for experiments with small population sizes and weighted (asymmetric) (and comparable well-mixed) migration. For each statistical analysis, rate of increase (r) and final frequency of the beneficial mutant (K) are shown. In the case of K, results from both the final day of experimental data and nls models are compared.

Low population size:

Migration rate	Simple slope contrasts (STARx - WM)* Time	Estimate	SE	DF	t-ratio	p-value
1000 cfu/ml	In>Out	-0.104	0.03<br>67	99	-2.841	0.0055

Supplementary table 6: Statistical analyses of full models for experiments with weighted (asymmetric) migration and small population size. For each statistical analysis, the estimated analyzed slope contrast for the GLMM, standard error (SE), degrees of freedom, t-ratio, and p value are shown.
